# Targeting Cellular Senescence: Pathophysiology in Multisystem Age-Related Diseases

**DOI:** 10.3390/biomedicines13071727

**Published:** 2025-07-15

**Authors:** Jinxue Liu, Hongliang Yu, Yuanyuan Xu

**Affiliations:** Key Laboratory of Zoonosis Research, Ministry of Education, College of Animal Science, Jilin University, Changchun 130062, China; m15043115440@163.com (J.L.); 18104304931@163.com (H.Y.)

**Keywords:** cellular senescence, mechanisms, SASP, cardiovascular disease, neurodegenerative disease, musculoskeletal disease, prevention–treatment

## Abstract

With the intensification of global aging, the incidence of age-related diseases (including cardiovascular, neurodegenerative, and musculoskeletal disorders) has been on the rise, and cellular senescence is identified as the core driving mechanism. Cellular senescence is characterized by irreversible cell cycle arrest, which is caused by telomere shortening, imbalance in DNA damage repair, and mitochondrial dysfunction, accompanied by the activation of the senescence-associated secretory phenotype (SASP). In this situation, proinflammatory factors and matrix-degrading enzymes can be released, thereby disrupting tissue homeostasis. This disruption of tissue homeostasis induced by cellular senescence manifests as characteristic pathogenic mechanisms in distinct disease contexts. In cardiovascular diseases, senescence of cardiomyocytes and endothelial cells can exacerbate cardiac remodeling. In neurodegenerative diseases, senescence of glial cells can lead to neuroinflammation, while in musculoskeletal diseases, it can result in the degradation of cartilage matrix and imbalance of bone homeostasis. This senescence-mediated dysregulation across diverse organ systems has spurred the development of intervention strategies. Interventional strategies include regular exercise, caloric restriction, senolytic drugs (such as the combination of dasatinib and quercetin), and senomorph therapies. However, the tissue-specific regulatory mechanisms of cellular senescence, in vivo monitoring, and safety-related clinical translational research still require in-depth investigation. This review summarizes the progress in pathological mechanisms and interventions, providing theoretical support for precision medicine targeting senescence, which is of great significance for addressing health challenges associated with aging.

## 1. Introduction

Aging refers to the phenomenon of gradual decline in physiological functions during the life process. As time progresses, structural and functional changes occur in tissues and organs, leading to a reduced ability of the body to adapt to the external environment and weakened viability. This process is extremely complex and is caused by the combined action of multiple factors [1]. To date, it encompasses fourteen interconnected hallmarks of aging: genomic instability, telomere attrition, epigenetic alterations, loss of proteostasis, disabled autophagy, deregulated nutrient sensing, mitochondrial dysfunction, cellular senescence, stem cell exhaustion, altered intercellular communication, chronic inflammation, and dysbiosis, psychosocial isolation, and extracellular matrix changes [2]. Cellular senescence is considered a core hallmark of aging, during which cells cease to divide and enter a state of permanent growth arrest [3].

Senescent cells are characterized by their inability to proliferate and enhanced activity of anti-apoptotic pathways. They release various chemokines, cytokines, growth factors, and proteases, a phenomenon termed the senescence-associated secretory phenotype (SASP) [4]. Although the mechanisms of cellular senescence in tissues are not fully understood, the evidence suggests an association between the accumulation of senescent cells and chronic inflammation [5]. This chronic inflammation can be destructive and lead to tissue damage. Moreover, it is noted that during the aging process, the body’s susceptibility to diseases increases markedly, with frequent occurrences of various conditions, including cardiovascular, musculoskeletal, cancer, and neurological diseases.

Scientists have long suspected an overlooked but crucial link between aging and many chronic human diseases. Aging increases the risk of many common diseases [3]. This article focuses on the close and complex association between cellular senescence and age-related diseases, aiming to outline the intricate connections between the two comprehensively. It seeks to deeply analyze the mechanisms by which cellular senescence influences the development of various diseases, including cardiovascular diseases, neurodegenerative disorders, and metabolic conditions. In-depth research into the mechanisms of cellular senescence not only reveals the fundamental pathogenesis of age-related diseases but also provides extensive opportunities for developing novel therapeutic strategies. This work thus offers a detailed theoretical basis for subsequent research, facilitating the exploration of more effective prevention and treatment strategies for age-related diseases.

## 2. Cellular Senescence

In recent years, the escalating proportion of the global aging population has spurred intensive investigations into the mechanisms of aging, driving increasingly comprehensive research on cellular senescence, ranging from in-depth explorations of the senescence-associated secretory phenotype (SASP) to the elucidation of its roles in specific diseases.

The term “cellular senescence” was first proposed over six decades ago, when Hayflick and Moorhead described that primary cells cultured in vitro exhibit a finite capacity for passaging, in which they are unable to proliferate indefinitely in culture, a characteristic that stands in stark contrast to the unlimited replicative features of cancer cells [6]. When cells reach their replicative limit, the number of their replications is termed the Hayflick limit [6]. Cellular senescence primarily refers to the state in which, when cells respond to stress and damage, their cell cycle enters an irreversible state of arrest [7]. Additionally, cellular senescence also serves as a tumor escape mechanism; for example, GADD45G-induced senescence in hepatocellular carcinoma cells inhibits the growth of tumor cells [8]. However, some senescent cells have been found to be carcinogenic; therefore, the roles of senescent cells cannot be explained by a single simple perspective.

To date, the significance of cellular senescence remains highly controversial. Some studies have found that cellular senescence plays a role in normal physiological processes [4], for instance, conferring certain benefits to embryonic development and organismal damage repair. Additionally, the evidence demonstrates that cellular senescence acts as a contributor to organismal aging and is closely associated with numerous age-related diseases [4]. One advancement in the research on cellular senescence is the discovery of senescence markers, such as β-galactosidase, P16, P21, etc. However, no single marker is applicable to all types of cells. Unique cellular senescence markers in vivo are still under investigation.

**Figure 1:**
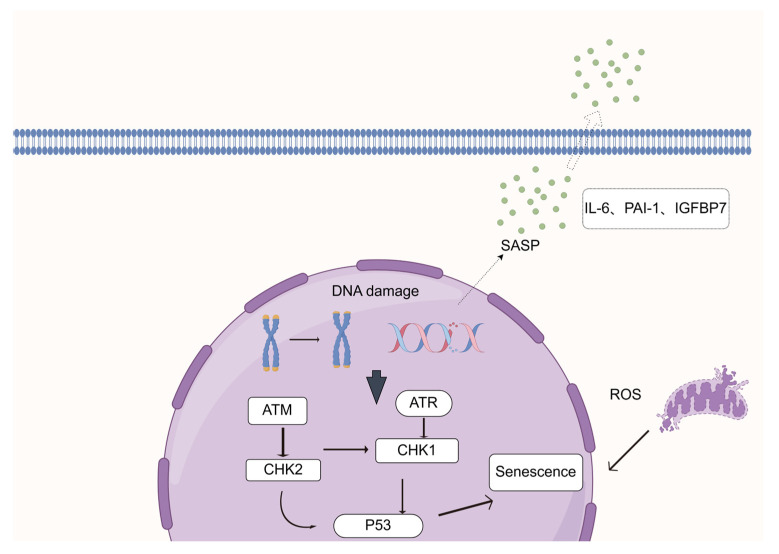
In the intracellular milieu, reactive oxygen species (ROS) can induce DNA damage, perturbing the normal DNA architecture. (As shown in the diagram, ROS acts on the cell to bring about this initial step.) This damage triggers the activation of the ATR (Ataxia Telangiectasia and Rad3-related) protein kinase. The activated ATR then acts on the tumor suppressor protein P53 via downstream signaling cascades involving phosphorylation-dependent molecular relays (matching the pathway where DNA damage activates ATM/ATR, which signal through CHK2/CHK1 to P53 in the diagram). Functioning as a pivotal signaling hub, P53 integrates these inputs to initiate transcriptional programs driving cellular senescence (corresponding to P53 and leading to the “Senescence” outcome in the figure). Additionally, extracellular factors of the senescence-associated secretory phenotype (SASP), including IL-6, PAI-1, and IGFBP7, as depicted, further mediate bidirectional crosstalk between senescent cells and their microenvironment, collectively modulating the senescence cascade (aligning with the SASP being secreted from the cell and interacting with the outside as shown). This figure was created using FigDraw 2.0. Thanks to FigDraw 2.0.

Current research on cellular senescence focuses on its core mechanisms and complex effects. Telomere shortening, as a classical molecular mechanism, triggers cell cycle arrest by affecting chromosomal stability and activating pathways such as p53/p21 and p16. DNA damage, particularly double-strand breaks (DSBs), acts as another critical inducer, with an imbalance in its repair and chromosomal instability collectively driving the progression of cellular senescence. However, the senescence-associated secretory phenotype (SASP) exhibits functional duality: it participates in cancer suppression, tissue repair, and immune regulation by secreting proinflammatory cytokines, growth factors, etc., while also potentially inducing chronic inflammation and tissue damage due to excessive accumulation. These mechanisms interweave to demonstrate the multidimensional roles of cellular senescence in physiological homeostasis and disease development, yet SASP’s specific in vivo markers and precise regulatory networks still await in-depth clarification. Cellular senescence, driven by DNA damage and regulated via p53 signaling, triggers SASP secretion, fueling inflammation and tissue dysfunction (as shown in Figure 1). This schematic maps the molecular cascade of senescence, highlighting its role in age-related pathologies. Below, we explore these mechanisms and potential interventions to combat senescence-linked diseases.

### 2.1. Telomere Shortening

Telomeres are nucleoprotein structures composed of tandem repetitive sequences at the ends of chromosomes and a protein complex called shelterin, which play a crucial role in chromosome replication and stability [9]. In mammals, the tandem repetitive ends of telomeres are composed of TTAGGG repeats, with the 3′ end being a G-rich sequence referred to as the G-prominent end. The G-prominent end invades the double-stranded DNA to form a D-loop, which, in turn, further forms a higher-order chromatin structure termed the T-loop [10,11]. This structure enables chromosome ends not to be recognized as double-strand breaks (DSBs), thereby maintaining genome stability and preventing the interconnection between chromosome ends. Shelterin, which binds to telomeres to provide further protection, is composed of six proteins: telomere repeat-binding factor 1 (TRF1), TRF2, repressor/activator protein 1 (RAP1), protection of telomeres 1 (POT1), TRF1-interacting nuclear protein 2 (TIN2), and TIN2 and POT1-interacting protein (TPP1) [12]. With each cell replication, the telomere length shortens by approximately 20 base pairs (bps). When shortened to a critical length, shelterin can no longer recognize and bind to telomeres. DNA repair mechanisms will then recognize such telomeres as double-strand breaks (DSBs). However, telomerase prevents this from happening; it is a DNA polymerase composed of a specific telomere reverse transcriptase (TERT) and telomere RNA (TER) [13,14]. However, most somatic cells lack telomerase activity.

Telomere shortening is not solely due to replicative shortening; it can also result from oxidative stress, nuclease processing, and telomere fragment loss [15,16,17]. Telomere shortening triggers ATM activation, which leads to the phosphorylation of Chk1/Chk2. This process thereby activates the p53/p21 pathway, resulting in cyclin-dependent kinase (CDK) inhibition and subsequent G1 phase arrest. In this context, phosphorylated histone H2AX (γH2AX) foci accumulate in senescent human fibroblasts. At these sites, γH2AX rapidly condenses and anchors DNA repair proteins—such as 53BP1, MDC1, and NBS1—proximal to telomere damage.

The formation of γH2AX foci is associated with the activation of DNA damage response effector kinases ATM, CHK1, and CHK2 [18]. However, γH2AX foci accumulation is not exclusively caused by telomere shortening; these foci also assemble at DNA double-strand breaks (DSBs) in senescent cells and cells derived from aged individuals. Surprisingly, certain mice with long telomeres and active telomerase still develop age-related pathologies. This phenomenon is attributed to an inherent vulnerability of telomeres to oxidative stress, a type of damage that accumulates with advancing age [15,19]. Among the target genes of p53, p21 is rapidly activated. In cultured fibroblasts, p21 does not persist, suggesting it may not be essential for senescence. In contrast to p21, p16 accumulates relatively slowly in senescent cells [20,21]. The inhibition of cyclin-CDK complexes by p21 and p16 leads to the sustained activation of the retinoblastoma protein (RB), which suppresses cell cycle progression by sequestering E2F family transcription factors [6].

### 2.2. DNA Damage Response

Among the multiple causes leading to cellular senescence, in addition to telomere damage, the most common is DNA damage. However, DNA damage response does not exist in all senescent cells [22].

DNA damage response in senescent cells is primarily categorized into persistent damage and double-strand breaks (DSBs). DSBs are considered the most deleterious form of DNA damage [23]. Several research groups have confirmed that the frequency of DSB-associated foci increases as cellular senescence progresses [24,25,26]. However, the presence of DSBs does not necessarily directly cause cell cycle arrest. While DSBs can impair cell division in certain contexts, most cells carrying DSBs still proliferate. Once DSBs arise in cells, a rapid and ordered response program is initiated to resolve them, preventing adverse effects on cellular function [27,28]. DSBs are primarily detected by protein complexes, most notably the MRE11/RAD50/NBS1 (MRN) complex and the Ku70/Ku80 heterodimer [23]. Cellular apoptosis or senescence occurs only when DSBs (double-strand breaks) in cells cannot be fully repaired. Even when DSBs are repaired, the best-case scenario of restoring the intact double helix may still introduce chromosomal mutations, leading to chromosomal instability. Telomere shortening and chromosomal instability are well-established primary drivers of cellular senescence [29].

### 2.3. The Senescence-Associated Secretory Phenotype (SASP)

Although cellular senescence is characterized by cell cycle arrest, cells remain metabolically active and secrete diverse components, including proinflammatory cytokines (e.g., IL-6 and IL-8), chemokines (e.g., monocyte chemoattractant proteins [MCPs] and macrophage inflammatory proteins [MIPs]), growth factors (e.g., transforming growth factor-β [TGF-β] and granulocyte–macrophage colony-stimulating factor [GM-CSF]), extracellular matrix components, enzymes (e.g., matrix metalloproteinases), and angiogenic factors, among others [28,29]. This behavior is termed the senescence-associated secretory phenotype (SASP), and many non-autonomous behaviors of senescent cells are mediated by the SASP [29,30].

Regardless of its original purpose, the senescence-associated secretory phenotype (SASP) exerts both beneficial and detrimental effects. SASP-mediated tumor suppressive functions, such as the secretion of interleukin-6 (IL-6), interleukin-8 (IL-8), plasminogen activator inhibitor-1 (PAI-1), and insulin-like growth factor-binding protein 7 (IGFBP7), enhance senescence arrest in in vitro experiments [31]. Moreover, the SASP (senescence-associated secretory phenotype) is a required factor for OIS (oncogene-induced senescence) in cultured HDFs (human dermal fibroblasts). The knockdown of IL-6 and miRNA screening assays consistently demonstrate the bypass of senescence [32,33,34].

Additionally, in certain liver-cancer animal models, the SASP contributes to the further formation of an anti-tumor microenvironment by inducing macrophage polarization [35]. The in vivo impact of the SASP on the tissue microenvironment is contingent upon multiple determinants, including the cell types undergoing cellular senescence (e.g., stromal or epithelial cells, with both normal and cancerous cells susceptible to senescence), the underlying etiology of senescence, and the activators of innate immune pathways governing the SASP (such as cGAS-STING signaling, Toll-like receptors [TLRs], etc.) [36]. The SASP not only enhances cellular senescence in an autocrine manner [33] but also mediates its paracrine effects.

SASP factors can remodel tissues in a paracrine manner, for instance, by altering the proliferative and migratory capacities of adjacent cells (such as stromal cells, immune cells, and cancer cells) [22,37,38]. Additionally, SASP factors are capable of stimulating angiogenesis while augmenting the immunosuppressive microenvironment [39,40]. Collectively, the contextual complexity of SASP-mediated effects on the tissue microenvironment underscores the critical need to identify cell populations secreting specific SASP factors for therapeutic targeting [37,41].

Senescent cells influence both themselves and the surrounding microenvironment through autocrine and paracrine pathways. Through autocrine signaling, cells reinforce their own senescence; via paracrine signaling, they secrete components of the SASP (senescence-associated secretory phenotype) to communicate with neighboring cells, thereby shaping the local microenvironment and stimulating adjacent cells to undergo senescence. This paracrine effect involves factors such as TGF-β family members, vascular endothelial growth factor (VEGF), and chemokines, among others [31,35,42], or trigger tissue repair. Additionally, senescent cells undergo transcriptional, epigenetic, and morphological changes.

It is noteworthy that the formation of the senescent microenvironment is inseparable from the role of the SASP (senescence-associated secretory phenotype). The senescent microenvironment is defined by several critical hallmarks: compromised fibroblast functionality, accumulation of senescent fibroblasts, disruption of extracellular matrix integrity, and induction of age-associated chronic inflammation [43,44,45]. The SASP plays a pivotal role in the formation of the senescent microenvironment. While senescent cells and SASP can exert transient beneficial effects—such as improving the regenerative capacity and stemness of keratinocytes via brief exposure to the SASP—they may become problematic with advancing age. Senescent cells tend to accumulate in bodily tissues, imposing harm on the organism [46]. Studies have demonstrated that the presence of the SASP is associated with degenerative diseases. Additionally, the SASP contributes to chronic inflammation and impedes tissue repair functions. Another detrimental effect of the SASP involves its paracrine influence, whereby neighboring cells undergo senescence conversion through paracrine signaling [47,48,49]. Eliminating senescent cells can reduce SASP production, thereby improving the prognosis of geriatric syndromes and aging-related diseases while enhancing organismal repair capacity [50].

In fact, the SASP is a fundamental factor behind many phenomena [51]. For example, senescent cells contribute to wound healing and fibrotic scar resolution by restoring homeostasis in fibrotic cells [35]. Furthermore, in response to damage stimuli, senescent cells induce the reprogramming of surrounding cells [52].

NF-κB is a transcriptional activator involved in the activation of numerous inflammatory cytokines. The induction of many components of the SASP (senescence-associated secretory phenotype) requires NF-κB. In certain animal models, the simultaneous knockout of p53 and NF-κB completely abrogates oncogene-induced senescence [53,54]. Alternatively, the siRNA-mediated knockdown of NF-κB also bypasses senescence [55]. If the production of SASP cytokines can stimulate the immune system to clear senescent cells, this could lead to tumor suppression that is as effective as apoptosis [42].

Numerous experiments have confirmed that the SASP (senescence-associated secretory phenotype) can induce immune cells, including macrophages, natural killer (NK) cells, and T cells. However, the number of senescent cells exists within a defined threshold; exceeding this threshold leads to their accumulation, thereby increasing tissue damage and contributing to the development of multiple diseases or immune dysregulation [47,56,57,58]. Cellular senescence has been demonstrated to serve as an anticancer mechanism under certain conditions. In numerous experiments, following chemotherapy, after tumors are eliminated, cellular senescence and the SASP occur [59,60]. In the RAS-activated mouse liver, secretion of the SASP elicits a robust immune response in the host. Activation of RAS in CD4+ cell-deficient mice, however, accelerates liver cancer development [61].

This also indirectly supports this theory. In SASP experimental reports, senescent cells are shown to secrete a large number of cytokines, including proinflammatory factors such as interleukin-6 (IL-6) and interleukin-8 (IL-8). The persistent presence of these proinflammatory factors leads to chronic inflammation, which serves as a pathogenic basis for many age-related diseases [62,63]. Chronic inflammation, distinct from acute inflammatory responses, is characterized by prolonged immune activation and tissue damage, contributing to the development of conditions such as cardiovascular disease, neurodegeneration, metabolic syndromes, and cancer. This highlights the dual role of the SASP: while it can mediate tumor suppression or tissue repair in acute contexts, its prolonged activity drives inflammatory pathologies associated with aging. Experiments conducted in 2011 and 2016 have demonstrated that the elimination of senescent cells reduces the levels of proinflammatory cytokines in the mouse body. This indicates that the SASP may contribute to inflammation [62,63].

On the other hand, in 2018, it was demonstrated that transplanting senescent cells into mice promotes organismal dysfunction and is sufficient to induce inflammation. Many mysteries still surround the role of senescent cells because the SASP exhibits multiple functions under different conditions and sometimes opposite functions [41].

The interwoven interactions of these mechanisms underscore the multifaceted character of cellular senescence as a complex biological process, one that represents both a protective response of the organism to injury and a potential causative factor in age-related pathologies.

Although the dissection of pathways such as telomere shortening, DNA damage response (DDR), and senescence-associated secretory phenotype (SASP) has outlined the molecular landscape of cellular senescence, the precise regulatory code governing its role in in vivo homeostasis, the clinical diagnostic value of specific senescence markers, and the development of targeted interventions for preventing and treating senescence-related diseases still require breakthroughs in two critical bottlenecks: the dissection of tissue-specific regulatory mechanisms and the spatiotemporal dynamic monitoring of senescence processes.

Future research is expected to leverage multidisciplinary integration and, on the basis of elucidating the heterogeneity of senescent cells, pave new avenues for senescence intervention and disease therapy.

Indeed, as a core biological event in the organismal aging process, cellular senescence also plays a critical role in age-related diseases. From the cardiovascular system to neurodegenerative and musculoskeletal diseases, cellular senescence continuously drives disease initiation and progression through distinct molecular pathways and cell–cell interaction patterns. Next, we focus on the fields of cardiovascular diseases, neurodegenerative diseases, and musculoskeletal diseases and conduct an in-depth dissection of the unique pathophysiological significance of cellular senescence in these contexts. This endeavor aims to provide a more comprehensive theoretical foundation for understanding the common mechanisms of senescence-related diseases and advancing the development of multidisciplinary research.

## 3. Cellular Senescence in Age-Related Diseases

With the accelerated progression of global population aging, age-related diseases have emerged as a significant challenge to human health. Cellular senescence, a core biological process underlying organismal aging, is profoundly involved in the pathogenesis and progression of multiple age-related conditions, including cardiovascular diseases, neurodegenerative disorders, and musculoskeletal diseases.

From cardiomyocyte senescence triggered by cardiometabolic dyshomeostasis to neuronal degenerative changes mediated by neuroinflammation and to functional decline of the musculoskeletal system caused by the disruption of bone homeostasis, cellular senescence drives disease progression through multiple mechanisms, such as metabolic dysfunction, inflammatory microenvironment remodeling, and protein stasis imbalance. In-depth exploration of the mechanistic roles of cellular senescence in age-related diseases not only aids in deciphering the fundamental laws governing disease development but also provides critical theoretical foundations and practical directions for developing innovative therapies targeting cellular senescence to improve patient outcomes.

### 3.1. Cellular Senescence in Cardiovascular Diseases

Over the past decade, emerging evidence has uncovered the association between cellular senescence and cardiovascular diseases (CVDs) [3]. As a primary cause of global mortality, World Health Organization (WHO) statistics indicate that 17.9 million deaths were attributed to cardiovascular diseases in 2019, comprising 32% of all global fatalities. Among these, 85% of deaths were precipitated by myocardial infarction and cerebrovascular accident (stroke). Thus, a thorough investigation into the role of cellular senescence in cardiovascular pathobiology is fundamental to elucidating the mechanistic underpinnings of disease pathogenesis and progression [64].

The impact of senescence on cells is profoundly influenced by cell type, tissue composition, and organ microenvironment, resulting in marked heterogeneity in their effects. The heart constitutes a complex architecture of diverse cell types, including cardiomyocytes, endothelial cells, fibroblasts, vascular smooth muscle cells (VSMCs), immune cells, and cardiac progenitor cells (CPCs) [65]. Extensive in vitro and in vivo investigations have demonstrated that all cardiovascular cell lineages undergo senescence during both physiological aging and cardiovascular disease (CVD) conditions [65]. Although senescent cells do not exhibit cell-type-specific morphological alterations, their morphology differs distinctly from normal cells, characterized by reduced selective permeability, increased membrane fragility, nuclear envelope breakdown, intracellular accumulation of lipofuscin, chromatin architectural changes, and degeneration of numerous intracellular structures and organelles [66,67,68,69,70].

The heart has the highest metabolic demand among all organs, accounting for approximately 8% of total energy expenditure despite comprising only 0.5% of body weight [3]. An early hallmark of the maladaptive heart is the loss of metabolic flexibility, whereby metabolic signals regulate transcription, translation, and post-translational signal transduction in the heart [71]. During senescence, cellular metabolic dysregulation occurs, which is at least partially associated with mitochondrial dysfunction, aberrant proteostasis, altered autophagic properties, and the presence of dysfunctional lysosomes, thereby leading to the accumulation of macromolecular damage [22,72].

Consequently, the role of cellular senescence in age-related CVDs likely stems from alterations across multiple cell types. Against this backdrop, this review aims to summarize senescent cells in two major types of cardiovascular disorders within the cardiovascular system and elaborate in detail on the pathophysiological implications of cellular senescence in cardiovascular diseases, with the goal of providing novel insights and theoretical foundations for the prevention and treatment of cardiovascular diseases.

#### 3.1.1. Cellular Senescence in Myocardial Infarction

Myocardial infarction (MI), one of the most prevalent cardiovascular diseases, exhibits a profound association between its pathological mechanisms and cellular senescence [73]. Ischemic injury induced by MI not only elicits structural and functional alterations in the heart but also triggers the senescence program in cardiomyocytes, a process confirmed as a critical driver of myocardial dysfunction [73]. Cardiac pump function is directly compromised by senescence: senescent cardiomyocytes display impaired contractility and disrupted conduction patterns, serving as major contributors to cardiomyopathy and arrhythmia [74]. Morphologically, senescent cardiomyocytes exhibit typical aging features, such as lipofuscin deposition and nuclear envelope breakdown, in addition to structural changes like increased cell size and morphological irregularities [66,75].

In addition to morphological changes, senescent cardiomyocytes exhibit the specific marker SA-β-Gal (senescence-associated β-galactosidase), a lysosomal enzyme that is readily detectable in situ and in vitro [76]. The positivity rate of SA-β-Gal is significantly elevated in naturally aged hearts [77], while MI in mice elicits multiplicative increases in the expression of senescence biomarkers, such as p16INK4a and p21CIP1 [78,79]. Notably, alterations in the senescence-associated secretory phenotype (SASP) of senescent cardiomyocytes are particularly pronounced: the released proinflammatory cytokines (e.g., TNF-α and IL-6) [77] and growth factors (e.g., GDF15 and TGF-β) collectively constitute the “senescence secretome” that promotes inflammatory cascades [77].

Senescent cardiomyocytes remodel the local microenvironment through the release of SASP factors, establishing a “senescence-inflammation” vicious cycle [80]. For instance, SASP factors, such as ANG II and ET-1, secreted by senescent endothelial cells directly induce senescence in neighboring cardiomyocytes [81,82,83]. Concurrently, the accumulation of chemokines, like CXCL10 and CCL5, in the post-myocardial infarction microenvironment [77] further recruits immune cells, exacerbating tissue damage. The molecular basis of this interplay lies in SASP factors sustaining inflammatory signal amplification via NF-κB pathway activation while suppressing the proliferative and differentiative capacities of cardiac progenitor cells [84].

Among the 14 recently proposed hallmarks of aging, “extracellular matrix (ECM) alterations” have been implicated in post-myocardial infarction fibrosis [85]. Senescent cells secrete proteases such as MMP3 (matrix metalloproteinase 3) to degrade ECM components, while TGF-β (transforming growth factor-β) promotes the transdifferentiation of fibroblasts into myofibroblasts, thereby reducing cardiac compliance [86]. This synergistic interaction between ECM remodeling and cellular senescence drives the progression from MI to heart failure [81,82,83].

#### 3.1.2. Cellular Senescence in Heart Failure

HF is a worldwide problem. Characterized by the heart’s inability to pump adequate blood to meet the body’s metabolic demands, HF is a life-threatening cardiovascular disorder that annually contributes to a substantial global mortality burden, particularly among patients aged over 50 years [39,87]. The progressive accumulation of senescent cells in cardiac tissue represents the key pathological basis of this phenomenon [50]. The heightened susceptibility of older adults to chronic heart failure (CHF) is intricately linked to prolonged exposure to injurious stressors, encompassing genomic, epigenetic, oxidative, autophagic, inflammatory, and regenerative insults, alongside the progressive accumulation of senescent cells within cardiac tissue [88]. The increased susceptibility of older adults to CHF essentially results from the combined effect of aging-related stresses, such as genomic damage, mitochondrial dysfunction, chronic inflammation, and metabolic remodeling of senescent cells [88]. Following prolonged exposure to oxidative stress and DNA damage stimuli, p21Cip1- and p53-positive senescent cells gradually accumulate in the heart, persistently disrupting the myocardial microenvironment through the secretion of proinflammatory cytokines and matrix-degrading enzymes [88,89].

While aging itself is not a direct etiology of HF, its induced cardiac adaptive changes (such as left ventricular hypertrophy and myocardial cell hypertrophy) and reduced β-adrenergic responsiveness collectively constitute the pathological basis for HF development [73]. Recent studies indicate that each 10% increase in senescent cell burden within the aging heart is associated with a 2.3-fold elevation in the incidence of diastolic dysfunction, a correlation particularly pronounced in HF with preserved ejection fraction (HFpEF) [90]. Vascular endothelial cells, as a key component of the blood–tissue barrier, play a dual role in the pathogenesis of HF. On the one hand, senescent endothelial cells exhibit typical features of functional decline, including increased arterial stiffness, impaired flow-mediated vasodilation, and intimal thickening due to inflammatory cell infiltration and lipid deposition [91]. On the other hand, senescent endothelial cells form a “senescence signaling cascade” through the release of endothelial microparticles (EMPs), membranous vesicles with a diameter of 50–1000 nm containing key factors for p53/p21 activation, which induce premature senescence in adjacent normal endothelial cells. This process is significantly accelerated in HF risk factors such as hypertension and diabetes [92].

Mechanistically, the pathological effects of senescent endothelial cells are mediated through three pathways: First, senescence induces the downregulation of endothelial nitric oxide synthase (eNOS) expression, accompanied by increased reactive oxygen species (ROS) production, both of which collectively impair vasodilatory function [93]. Second, the sustained release of endothelin-1 (ET-1) and transforming growth factor-β (TGF-β) promotes the transdifferentiation of vascular smooth muscle cells (VSMCs) into myofibroblasts, exacerbating vascular wall fibrosis [92]. Third, NF-κB signaling upregulates the expression of vascular cell adhesion molecule-1 (VCAM-1) and intercellular adhesion molecule-1 (ICAM-1), mediating monocyte recruitment to the vascular wall [94,95,96]. Additionally, senescent endothelial cells exhibit the proinflammatory senescence-associated secretory phenotype (SASP), including TNF-α, IL-1, IL-6, and IL-8 [97]. Elevated levels of SASP components correlate with the severity of HF, forming a “senescence-inflammation” positive feedback loop [94].

In the senescence-accelerated mouse (SAM) model, a high-fat and high-salt diet induces a 4.7-fold increase in the positivity rate of senescence markers (acetylated p53/CD31 double staining) in aortic endothelial cells, accompanied by elevated left ventricular end-diastolic pressure (LVEDP) and interstitial fibrosis. This phenotype closely mirrors the pathological features of human HF with preserved ejection fraction (HFpEF) [90].

The secretory phenotype of senescent cardiomyocytes (SASP) constitutes the “molecular driver” of HF progression: factors such as IL-6 and GDF15 activate the fibrotic program in fibroblasts, while TNF-α and MCP-1 recruit macrophages to exacerbate inflammatory responses [77].

The essence of cardiac aging is a pathological network formed by paracrine signaling across multiple cell types: ANG II and ET-1 secreted by senescent endothelial cells induce cardiomyocyte senescence, whereas IL-6 released by senescent cardiomyocytes activates the p38 MAPK pathway in fibroblasts, promoting collagen deposition [80]. This “cross-activation” phenomenon is particularly pronounced in metabolic dysfunction. Under diabetic conditions, the sustained release of SASP factors mediated by metabolic memory serves as the molecular basis for chronic activation of the “senescence-inflammation” axis in diabetic cardiomyopathy [98].

#### 3.1.3. Other Types of Cellular Senescence in Cardiovascular Diseases (CVDs)

Cardiac fibroblasts, as the primary executors of extracellular matrix (ECM) remodeling, play a decisive role in cardiac fibrogenesis through their senescence [98]. Fibroblasts manifest senescent traits via classical cell cycle arrest and a proinflammatory secretory phenotype (SASP) [78]. In aged patients and mouse models, senescent fibroblasts are responsible for the gradual deposition of ECM in the heart, leading to cardiac fibrosis and dysfunction [99].

Cardiac progenitor cells (CPCs), serving as the core population for endogenous cardiac repair, exhibit senescence that directly leads to the age-related decline in myocardial regenerative capacity. CPCs isolated from the hearts of older adults aged 76 to 86 show significantly shortened telomeres, high expression of p16INK4a, and enhanced SA-β-Gal (senescence-associated β-galactosidase) activity. These senescent features are colocalized with the DNA damage markers, γ-H2AX foci [100].

Cardiac progenitor cells are multipotent and self-renewing cell populations within the myocardium [101,102]. Studies have demonstrated that CPCs isolated from adults aged 76 to 86 exhibit typical senescent features: markedly shortened telomeres, increased p16Ink4a expression, enhanced SA-β-gal activity, and elevated DNA damage markers [100]. Vascular smooth muscle cells (VSMCs), responsible for extracellular matrix synthesis, arterial contraction regulation, and injury repair, reside adjacent to the endothelium and are susceptible to circulating toxins, inflammatory mediators, and blood pressure fluctuations [92,103]. Senescent VSMCs display characteristics such as SA-β-gal expression, telomere shortening, p16Ink4a upregulation, accompanied by growth arrest and SASP (senescence-associated secretory phenotype) formation, thereby recruiting inflammatory and immune cells to the vascular wall [104]. Their specific senescence can trigger acute ischemic cardiovascular events (e.g., occlusion of moderately stenosed vessels) and increase the risk of adverse clinical outcomes, such as severe heart failure [105,106].

Natural Killer (NK) cells serve as the core of immune surveillance against senescent cells, yet senescent NK cells exhibit diminished cytotoxicity and cytokine-secreting capacity, escalating the risk of infections and inflammation. Their functional impairment represents a critical contributor to coronary heart disease and CHF [107].

Cellular senescence significantly impairs the ability of macrophages to clear phagocytose-damaged cardiomyocytes and maintain homeostasis, driving their phenotypic shift toward a proinflammatory state. This is characterized by the increased expression of matrix metalloproteinases (MMPs) [108]. Senescent macrophages release proinflammatory cytokines, such as IL-4 and IL-13, which induce cardiomyocyte hypertrophy via the phosphorylation of signal transducer and activator of transcription 3 (STAT3). Sustained hypertrophy leads to maladaptive ventricular remodeling, a primary etiology of CHF [99].

Collectively, these studies demonstrate that cellular senescence primes immune cells to augment disease risk through diverse mechanisms. These findings illuminate the pathophysiological significance of cellular senescence in cardiovascular diseases, providing critical references for subsequent investigations.

### 3.2. Cellular Senescence in Neurodegenerative Diseases

Neurodegenerative diseases are a group of heterogeneous brain disorders [109]. As is well known, aging is the main risk factor for neurodegeneration, and the most common neurodegenerative diseases are observed in older adults [110].

Increasing evidence indicates that cellular senescence is involved in the development of neurodegenerative diseases (NDs). The formation of senescent cells accelerates organ-aging processes and functional decline and promotes the occurrence of different NDs [111].

Senescence is a programmed change in cell state induced by various stresses, in which mitotic cells stop dividing but do not die. However, senescence also affects post-mitotic neurons, manifested as DNA damage (a mediator of senescence), mitochondrial dysfunction, the senescence-associated secretory phenotype (SASP), increased levels of senescence-associated β-galactosidase, nuclear morphological changes, accumulation of macromolecular aggregates, and increased levels of various cell cycle inhibitory proteins. Neurons are particularly vulnerable to senescence because they cannot dilute damaged sites through mitosis [112]. Neuronal senescence increases with age and can occur in the brain without obvious disease. However, senescence of neurons and glial cells may also be exacerbated by the disease and its subsequent effects on cells [113]. The accumulation of senescent cells and the occurrence of chronic inflammation, resulting from the release of the senescence-associated secretory phenotype (SASP), are the fundamental mechanisms by which senescent cells promote the aging process and the development of age-related diseases [114].

This research is dedicated to exploring the mechanisms and impacts of cellular senescence in neurodegenerative diseases, with a particular focus on Alzheimer’s disease (AD) and Parkinson’s disease (PD). As the most prevalent and extensively studied neurodegenerative disorders, AD and PD exhibit a particularly close association with cellular senescence during their pathological progression, characterized by prominent and representative senescence-related pathological features. In contrast, while cellular senescence has also been observed in other neurodegenerative diseases, such as Huntington’s disease (HD) and amyotrophic lateral sclerosis (ALS), the current research primarily remains at the level of common aging characteristics, and their specific molecular mechanisms await further exploration. The following sections systematically elaborate on the specific effects of cellular senescence in various neurodegenerative diseases and comprehensively summarize the major potential molecular mechanisms, aiming to clearly demonstrate the critical role of cellular senescence in disease progression.

#### 3.2.1. Cellular Senescence in Alzheimer’s Disease (AD)

Cell senescence plays a pivotal role in the pathological progression of AD. AD is the most prevalent neurodegenerative disorder in humans, characterized by amyloid plaques containing Aβ peptides and aggregates of neurofibrillary tangles composed of hyperphosphorylated or misfolded tau proteins [115]. Recent evidence indicates that Aβ-driven cellular senescence represents a complex cellular response mechanism in AD progression [116]. Senescent cells not only exacerbate Aβ pathology but also trigger neuroinflammatory cascades through the release of inflammatory factors [62].

Genomic analyses have revealed multiple genes associated with AD risk, which are linked to innate immune function and elevated inflammatory markers [117]. The neuroinflammatory hypothesis posits that microglia and astrocytes, as cells with distinct functional roles and mutual regulatory effects in brain tissue [118], when experiencing functional deficits, hinder the clearance of amyloid-β. Studies have shown that cell types prone to senescent features during normal aging also exhibit significant alterations in AD patients [118], senescent microglia and astrocytes trigger chronic neuroinflammation, leading to neuronal death, which further corroborates the synergistic role of cellular senescence and neuroinflammation in AD pathogenesis.

##### Microglia

Microglia, as the primary immune cells of the brain, originate from the bone marrow during development and functionally resemble macrophages [119]. In the normal state, microglia typically remain in a quiescent state, characterized by small cell bodies and highly branched processes [120]. However, during aging, they undergo significant changes, exhibiting a cellular senescence phenotype marked by cytoplasmic degeneration [120]. In vitro studies have shown that cultured microglia exposed to repeated stress stimuli (such as lipopolysaccharide administration) display typical senescence responses, including growth arrest, enhanced β-galactosidase activity, and senescence-associated heterochromatic foci [120].

Microglial senescence is closely associated with neuroinflammation and Alzheimer’s disease (AD) pathology. Microglia from aged mice produce large quantities of proinflammatory cytokines IL-6, IL-1β, and TNF-α [121], which are significantly upregulated in senescent cells [122]. More importantly, microglial senescence leads to immunological dysfunction, significantly reducing extracellular tau clearance efficiency while promoting tau phosphorylation and accelerating tau pathology spread [123]. In a neuronal tauopathy mouse model, depletion of senescent microglia effectively reduced tau pathology and significantly improved cognitive function [123], directly confirming the critical driver role of microglial senescence in AD progression [124].

##### Astrocytes

Astrocytes, as essential components of the central nervous system, participate in synaptic neuronal function and plasticity regulation, serving as the foundation for defense and regeneration [120]. With aging, their structure and function undergo significant changes: substantial accumulation of lipofuscin in the cytoplasm, and increased numbers of glial fibrillary acidic protein (GFAP) and vimentin filaments in aged animals [120], these changes are typical features of cellular senescence, suggesting the presence of a senescence-associated secretory phenotype (SASP) [120].

At the metabolic level, senescent astrocytes exhibit metabolic remodeling, with enhanced oxidative metabolism with age, limiting their ability to provide metabolic substrates for neurons [124]. In vitro studies have shown that cultured astrocytes under oxidative stress or ionizing radiation display characteristics of cellular senescence, including upregulated expression of senescence biomarkers [120], and senescent astrocytes downregulate genes associated with cell activation [125].

In the course of Alzheimer’s disease (AD), astrocyte senescence plays a key role. Senescence markers are significantly upregulated in astrocytes of AD patients, and β-amyloid (Aβ) can induce astrocyte senescence in vitro through reactive oxygen species (ROS), accompanied by upregulated expression of p38, IL-6, and IL-8 [126]. Astrocytes cultured from AD patients, compared with age-matched controls, not only show significantly increased expression of the key senescence molecule CDKi p16INK4A but also markedly elevated expression of matrix metalloproteinase MMP1, further confirming the close association between astrocyte senescence and AD pathological development [126].

#### 3.2.2. Cellular Senescence in Parkinson’s Disease

PD is the second most common neurodegenerative disorder, characterized by loss of motor control due to the degeneration and loss of dopaminergic neurons in the substantia nigra [127]. Compared with age-matched controls, senescence markers p16 and SASP factors (IL-6, IL-1α, IL-8, and MMP-3) are elevated in brain tissues of PD patients [127]. These findings suggest that cellular senescence is associated with dopaminergic neurodegeneration in PD.

##### Microglia

In Parkinson’s disease (PD), cellular senescence is closely linked to abnormal microglial activation-accumulation of activated microglia has been observed in both PD patients and mouse models, directly leading to a significant increase in local concentrations of inflammatory mediators and reactive oxygen species (ROS) [128]. Prolonged chronic inflammation further drives the functional shift of microglia toward a neurodegenerative phenotype, which essentially represents the pathological manifestation of cellular senescence in central immune cells [129]. Studies have confirmed that age-related elevation of peripheral and neuroinflammatory levels is not only a typical feature of cellular senescence but also directly promotes the pathological process during the prodromal stage of Parkinson’s disease [130]. Additionally, postmortem samples from the substantia nigra and basal ganglia of PD patients show significantly increased colocalization of α-synuclein deposits with activated microglia, a spatial distribution characteristic that suggests an interaction between protein aggregation and immune activation in the senescent cellular microenvironment [131]. These human pathological findings are corroborated by in vitro studies of mouse microglia: nitrated α-synuclein aggregates can trigger senescent damage to dopaminergic neurons by inducing an oxidative stress microenvironment [132].

These findings collectively indicate that microglial senescence plays a pivotal role in the PD pathological process by establishing a positive feedback loop of “inflammation-senescence-neurodegeneration”. Therapeutic strategies targeting the elimination of senescent microglia may thus provide a novel direction for PD intervention.

##### Astrocytes

Both PD patients and mouse models exhibit abnormal accumulation of activated astrocytes [128], and postmortem PD brain samples further confirm that these activated cells are accompanied by significantly enhanced senescent phenotypes [111]. This population of senescent astrocytes serves as key drivers of the local inflammatory microenvironment—not only promoting a sharp increase in concentrations of inflammatory mediators and reactive oxygen species (ROS), but also initiating neurotoxic cascades through overexpression of S100b protein [133].

Autopsy analyses of the substantia nigra in PD patients reveal particularly significant upregulation of S100b: this protein specifically induces astrocytic expression of inducible nitric oxide synthase (iNOS), thereby triggering explosive production of nitric oxide (NO) and superoxide radicals [134]. This mechanism has been validated in both PD patients and MPTP-induced mouse models-senescent astrocytes in the substantia nigra pars compacta (Snpc) continuously produce high concentrations of iNOS, and the free radicals catalyzed by iNOS can directly penetrate the blood-brain barrier or indirectly trigger programmed death of dopaminergic neurons via oxidative stress [135].

Notably, the functional relevance of senescent astrocytes to sporadic PD pathogenesis has been validated: when senolytic therapies reduce senescent astrocytes in PD mouse models, the degree of neurodegeneration is significantly alleviated. This finding not only confirms the causal role of astrocyte senescence in PD pathology but also reveals the central position of the “senescence-inflammation” interaction network in neurodegenerative diseases—the vicious cycle between proinflammatory factors and senescent cells represents the key pathological hub driving PD progression

#### 3.2.3. Cellular Senescence in Other Neurological Disorders

Through systematic evaluation of cellular senescence signatures in Parkinson’s disease (PD) and Alzheimer’s disease (AD), a noteworthy phenomenon has emerged: patterns of senescence-associated phenotypes—including the types of senescent cells involved—exhibit pathological specificity of significant research value. This specificity is not only observed in these two common diseases but also manifests as distinct senescence imprints in other neurodegenerative disorders.

Huntington’s disease, a progressive neurodegenerative disorder characterized by cognitive deficits, emotional lability, and motor dysfunction, exhibits a deep-seated association between its pathogenic mechanism and cellular senescence [136]. Studies have confirmed that mutant huntingtin protein in HD directly participates in inducing cellular senescence programs, and its abnormal accumulation disrupts key cellular processes such as DNA repair, oxidative stress regulation, and proteostasis [136]. This disruption leads to rapid accumulation of senescent cells in HD, and the accompanying proinflammatory senescence-associated secretory phenotype (SASP) triggers chronic neuroinflammation, further accelerating neurodegeneration and forming a vicious cycle of “protein pathology-cellular senescence-inflammatory activation” [137].

In the progressive motor neuron disease amyotrophic lateral sclerosis (ALS), the pathological role of cellular senescence is equally prominent [138,139]. ALS-derived astrocytes show high-level expression of senescence markers such as p21 and p16, while analysis of patient brain samples reveals significantly upregulated expression of senescence-related genes like p16INK4a and p21CIP1/WAF1 in glial cells and astrocytes [140]. Similarly, in a rat model carrying the ALS-related SOD1G93A mutation, microglia exhibit typical senescent features: enhanced SA-β-Gal activity, increased expression of p16INK4a and p53, accompanied by release of SASP factors such as matrix metalloproteinase-1 (MMP-1) and nitrotyrosine, confirming the central role of glial senescence in motor neuron injury [140].

It is noteworthy that pathological involvement of senescent cells is not limited to the above classic diseases. For example, HIV patients often experience premature aging-related complications, where the occurrence of HIV-associated neurocognitive disorder (HAND) is closely linked to cellular senescence in the central nervous system—viral infection can induce abnormal activation of senescence programs in neurons and glia by activating inflammatory signals and oxidative stress, thereby affecting neurological function [139].

In summary, cellular senescence plays a pivotal role in the progression of neurodegenerative diseases. The specific molecular mechanisms it exhibits in different diseases not only deepen our understanding of the pathological essence of these diseases but also provide a clear direction for the development of innovative therapeutic strategies. Interventions targeting cellular senescence (such as senolytic therapies) are expected to break the vicious cycle of “senescence-inflammation-neurodegeneration”, offering new therapeutic opportunities to improve patient outcomes.

### 3.3. Cellular Senescence in Musculoskeletal Diseases

As a dynamic organ, the skeleton relies on the coordination of multiple cell types to maintain bone homeostasis—the precise balance between bone formation and resorption under normal physiological conditions—with cellular senescence emerging as a key factor disrupting this balance: senescent osteoblasts and osteoclasts aberrantly regulate bone turnover rates, leading to bone mass loss [141].

The musculoskeletal system not only supports body movement and participates in blood production but also exhibits endocrine functions; however, with aging, cellular senescence-induced bone homeostasis imbalance gradually emerges, eventually leading to degenerative diseases such as osteoporosis and osteoarthritis, which severely affect the quality of life in older adults. This imbalance essentially results from senescent cells secreting SASP factors, disrupting osteoblast-osteoclast coupling [142]. Although drugs like bisphosphonates and denosumab have been used clinically, their benefits remain limited due to side effects or the physical condition of older adult patients, shifting research focus to cellular senescence—as a common pathological basis for age-related skeletal diseases, its mediated inflammatory microenvironment and matrix degradation offer a new rationale for targeted therapy [143,144]. Skeletal development and regeneration rely on the coordination of multiple signaling pathways mediating bone remodeling, yet cellular senescence induces dysfunction in these pathways, further compromising skeletal system stability [145].

The following will deeply analyze the molecular action pathways of cellular senescence in diseases such as osteoporosis and osteoarthritis from the perspective of tissue-specific effects of cellular senescence, providing a theoretical basis for the development of senescence-targeted therapies.

#### 3.3.1. Cellular Senescence in Osteoarthritis Inflammation

OA an age-related degenerative cartilage disease, is closely associated with cellular senescence in its core pathological mechanism [146]. As the only cell type in articular cartilage, chondrocytes maintain extracellular matrix homeostasis by synthesizing type II collagen, endowing cartilage with tensile strength; however, with aging, chondrocytes gradually undergo senescence, serving as a key driver in the development of OA.

Chondrocytes are crucial for cartilage tissue homeostasis, with cell loss or dysfunction representing the primary inducer of cartilage failure [147]. Their structure continuously changes during skeletal development and aging, while senescent chondrocytes exhibit unique characteristics: when chondrocytes senesce, a large quantity of proinflammatory mediators are produced in the joint, activating matrix metalloproteinases (MMPs) to degrade the cartilage extracellular matrix (ECM) [148]. Concurrently, synovial macrophages, fibroblasts, and osteoclasts synergize in the senescent microenvironment to promote the senescence-associated secretory phenotype (SASP), triggering inflammatory responses, altering subchondral bone remodeling, and accelerating cartilage loss and disease progression [149,150].

Research evidence further corroborates the close link between cellular senescence and OA: in aged mouse models, joint cartilage scores significantly correlate with key SASP factors (such as inducible nitric oxide synthase, MMP-13) [151], among these, MMP-13 is regarded as a critical mediator of OA inflammation [152]. The persistent inflammatory microenvironment induced by senescent cells not only causes chronic inflammation but also promotes senescence of adjacent healthy cells, exacerbating extracellular matrix degradation and synovitis, thus forming a vicious cycle [153]. Through comprehensive analysis of OA datasets and human aging databases, Geng et al. found that the senescence-related gene FOS is significantly upregulated in lesional tissues, genetically revealing the central role of cellular senescence in OA [154].

In summary, the continuous accumulation of senescent cells in joints, by altering the extracellular matrix and increasing mechanical stress, ultimately leads to biomechanical dysfunction and significantly elevates the risk of OA [153]. Cellular senescence pervades the entire pathological process of OA, emerging as a key target for understanding and intervening in this disease.

#### 3.3.2. Cellular Senescence in Osteoporosis

OP, a common systemic skeletal disease characterized by disruption of bone tissue microstructure and a decrease in bone mineral density, significantly increases the risk of fractures. Growing research has focused on cellular senescence, uncovering its critical role in the pathological process of OP [155].

In recent years, cellular senescence has been demonstrated to disrupt the balance of bone turnover through the release of secretory factors, which not only inhibit bone formation but also enhance bone resorption, thus exacerbating skeletal fragility [156]. Cellular senescence also exerts a remarkable impact on fracture healing, particularly in older adults, often leading to delayed healing accompanied by a reduction in mesenchymal progenitor cells (MPCs) [157]. Research data corroborate this phenomenon: the number of senescent cells in the fracture callus of aged mice increases drastically compared to young mice and non-fractured sites in aged mice [158].

During normal aging, changes in osteoblasts cannot be overlooked. Their quantity gradually decreases, accompanied by typical senescent manifestations, such as DNA damage, prominent senescence-associated secretory phenotype (SASP) features, and increased p21Cip1 expression [159]. Substantial evidence indicates that senescent osteoprogenitor cells, osteoblasts, and osteocytes are key drivers of aging-related bone physiological changes [160,161]. Experiments in the INK-ATTAC mouse model provide direct evidence: the genetic depletion of senescent osteocytes and osteoblasts alleviates age-related bone loss, further confirming the promoting role of cellular senescence and the SASP in OP development [162].

Collectively, these studies highlight the central role of senescent cells in age-related bone loss and OP. Senescent myeloid cells and osteocytes represent major sources of the SASP in the bone microenvironment, driving alterations in bone remodeling. Osteocyte senescence disrupts bone metabolism, whereas osteocyte depletion induces severe skeletal disorders, such as OP. Additionally, senescent osteoblasts contribute to the progression of OP. Thus, the accumulation of senescent cells exerts a profound impact on skeletal health and accelerates bone aging.

As a critical biological phenomenon in the course of life, cellular senescence is intricately intertwined with the occurrence and progression of cardiovascular diseases, neurodegenerative disorders, and musculoskeletal diseases. In the cardiovascular system, senescence of various cell types—from MI to HF—accelerates disease deterioration through mechanisms such as metabolic dysregulation and remodeling of the inflammatory microenvironment. In the field of neurodegenerative diseases, senescence of microglia, astrocytes, and other glial cells triggers neuroinflammation and protein stasis imbalance, serving as key drivers in the progression of AD, PD, and other related conditions. Within the musculoskeletal system, senescent cells disrupt bone homeostasis and erode cartilage tissue, exacerbating the risk of OP and OA.

**Figure 2:**
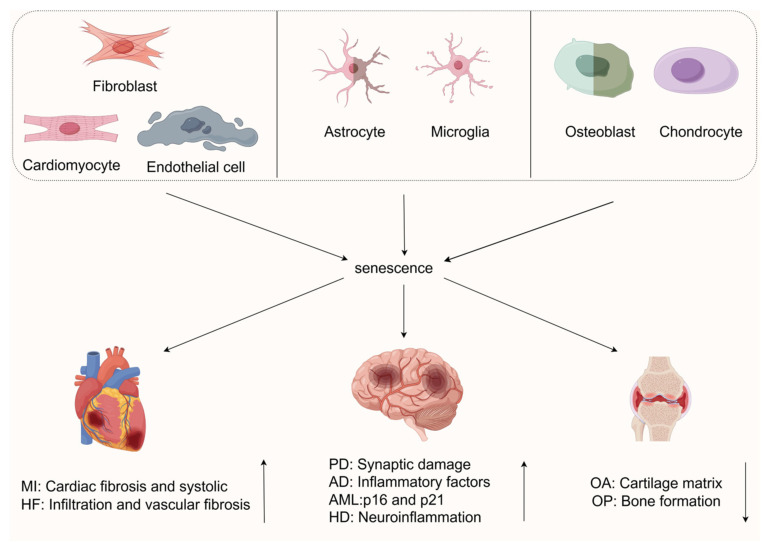
As shown, diverse cells (fibroblasts, cardiomyocytes, etc.) undergo senescence. In cardiovascular system, myocardial and endothelial cell senescence causes fibrosis and heart failure. In nervous system, neuron and microglial senescence worsen neurodegeneration (PD, AD, etc.). In musculoskeletal system, chondrocyte senescence accelerates OA cartilage degradation, and osteoblast senescence hinders OP bone formation. Via SASP paracrine and cell cycle arrest, senescent cells drive tissue damage and age-related disease pathology across systems. This figure was created using FigDraw 2.0. Thanks to FigDraw 2.0.

These findings not only deepen our understanding of disease pathophysiology but also illuminate new directions for future medical research. The development of innovative therapies targeting cellular senescence and its associated pathways holds promise to break through the limitations of conventional treatments, bringing revolutionary breakthroughs to improve patient outcomes and quality of life. Additionally, this research provides new theoretical support and practical possibilities for humanity’s fight against major diseases. Cellular senescence is closely linked to pathophysiological changes in cardiovascular, neurological, and musculoskeletal systems (as shown in Figure 2). Senescent cells induce inflammatory microenvironments and functional decline via SASP secretion and other mechanisms. This section focuses on cross-system interactions, molecular mechanisms, and advances in strategies like senolytic clearance and SASP modulation, providing theoretical support for preventing age-related multisystem diseases.

## 4. Prevention and Treatment of Cellular Senescence

Against the backdrop of global aging, age-related diseases increasingly threaten human health, urging research into preventing cellular senescence and developing precision therapies. Exercise inhibits senescence by elevating PF4, increasing GLUT4, and preserving telomeres [163,164,165,166]. Caloric restriction extends telomeres, reduces inflammation, and reprograms macrophages via single-cell validated gene expression changes [167,168,169,170,171,172]. Targeting senescent cell anti-apoptotic pathways, senotherapeutics have advanced: first-generation agents target signaling pathways, while second-generation strategies (lysosomal activators, CAR-T, and SASP inhibitors) offer safer specificity [173,174,175,176]. Efficacy and safety require clinical evaluation as senotherapeutics accelerate for age-related disease treatment [176].

## 5. Conclusions and Future Prospects

This review focuses on the core topic of cellular senescence, systematically elaborating its biological mechanisms, role in age-related diseases, and potential intervention strategies, with the aim of revealing the close link between cellular senescence and human health.

At the mechanistic level, the review primarily describes core pathways such as telomere shortening, DNA damage, and the senescence-associated secretory phenotype (SASP), elucidating how these drive cell cycle arrest and impact organismal homeostasis. However, critical gaps in knowledge remain to be addressed. For example, the dynamic regulation of cellular senescence remains poorly understood. The spatiotemporal patterns of senescent cell evolution in vivo—including their roles in physiological processes, such as embryonic development and tissue repair—are largely unclear. The key unresolved questions include how tissue-specific microenvironments differentially modulate senescence progression, as well as the synergistic networks between core pathways (e.g., telomere shortening, DNA damage, and the SASP). These require systematic dissection using multi-omics technologies and in vivo tracing methodologies.

In terms of disease associations, while the pathological roles of cellular senescence in cardiovascular diseases, neurodegenerative diseases, and musculoskeletal diseases have been thoroughly explored, demonstrating a close correlation between cellular senescence and these conditions, the validation of causal relationships remains in its infancy. In the future, it will be necessary to establish more precise animal models and clinical cohorts, utilize gene-editing technologies to specifically eliminate or induce senescent cells, and combine techniques such as single-cell sequencing to deeply investigate the cause-and-effect logic between cellular senescence and disease development. This will help clarify the key driving nodes of senescent cells in the disease progression.

In terms of intervention strategies, this review summarizes research achievements in exercise, dietary regulation, and senolytic therapies, demonstrating multidimensional research directions for preventing and treating cellular senescence. However, clinical translational research of these strategies faces significant challenges: both the precision application of exercise/dietary interventions and the safety assessment of senolytic therapies require large-scale, long-term clinical trial data. Current interventions lack systematic evaluation criteria, necessitating the establishment of a multidimensional assessment framework spanning cellular and animal models to human trials. Particular attention must be paid to potential compensatory risks following senescent cell clearance and the long-term impacts on systemic metabolism and immunity. Future research should focus on developing highly effective and low-toxicity aging intervention strategies, accelerating the translation of basic research into clinical applications, and opening new pathways for delaying aging and preventing age-related diseases.
